# National Medical Convention

**Published:** 1845-10-01

**Authors:** 


*National Medical Convention.*—The New York State Medical Society at its last annual meeting, adopted the following preamble and resolution:

"*Whereas*, it is believed that a National Convention would be conducive to the elevation of the standard of Medical Education in the United States, and whereas, there is no mode of accomplishing so desirable an object, without concert of action on the part of the medical Societies, Colleges, and institutions of all the States, therefore,

*Resolved*, That the New-York State Medical Society earnestly recommend a National Convention of delegates from the Medical Societies and Colleges in the whole Union, to convene in the city of New York, on the first Tuesday in May, in the year 1846, for the purpose of adopting some concerted action on the subject set forth in the foregoing preamble."

Shall this plan of a convention be carried into effect? This is for the profession in the different States of the Union to decide. We hope measures have been taken to bring the subject before the Societies and Schools of the several States, so as to insure seasonable action upon it. In the meantime, it is desirable that the matters which will come before a convention be discussed throughout the country by means of medical periodicals, and the various local associations, in order that the general sentiments of the profession may be elicited, and properly represented by the delegates. None can doubt the propriety, nay, the urgent necessity for the adoption of some means to elevate the standard of medical education, and advance the dignity and usefulness of the profession. There seems no other course but for the profession to endeavor by its own action to effect objects so desirable to itself, and still more important to the public. We fervently hope that this movement will meet with general concurrence, and cordial cooperation. Much may probably be done by Medical Journals toward awakening interest in the measure, and we trust this duty will not be omitted. We are gratified to see in the New Orleans Journal a warm approval of it. The Editors say, "we look upon this as one of the most important moves ever proposed in regard to the profession in this country, and we sincerely hope the call will be responded to throughout the Union."

